# GADEN: A 3D Gas Dispersion Simulator for Mobile Robot Olfaction in Realistic Environments

**DOI:** 10.3390/s17071479

**Published:** 2017-06-23

**Authors:** Javier Monroy, Victor Hernandez-Bennetts, Han Fan, Achim Lilienthal, Javier Gonzalez-Jimenez

**Affiliations:** 1 Machine Perception and Intelligent Robotics group (MAPIR), Instituto de Investigación Biomedica de Malaga (IBIMA), Universidad de Malaga, 29071 Malaga, Spain; javiergonzalez@uma.es; 2 Applied Autonomous Sensor Systems, Örebro University, Fakultetsgatan 1, 70182 Örebro, Sweden; victor.hernandez@oru.se (V.H.-B.); han.fan@oru.se (H.F.); achim.lilienthal@oru.se (A.L.)

**Keywords:** gas dispersal, robotics olfaction, gas sensing, mobile robotics, Robot Operating System (ROS)

## Abstract

This work presents a simulation framework developed under the widely used Robot Operating System (ROS) to enable the validation of robotics systems and gas sensing algorithms under realistic environments. The framework is rooted in the principles of computational fluid dynamics and filament dispersion theory, modeling wind flow and gas dispersion in 3D real-world scenarios (i.e., accounting for walls, furniture, etc.). Moreover, it integrates the simulation of different environmental sensors, such as metal oxide gas sensors, photo ionization detectors, or anemometers. We illustrate the potential and applicability of the proposed tool by presenting a simulation case in a complex and realistic office-like environment where gas leaks of different chemicals occur simultaneously. Furthermore, we accomplish quantitative and qualitative validation by comparing our simulated results against real-world data recorded inside a wind tunnel where methane was released under different wind flow profiles. Based on these results, we conclude that our simulation framework can provide a good approximation to real world measurements when advective airflows are present in the environment.

## 1. Introduction

Environmental monitoring, gas emission surveillance and other gas-sensing related tasks have nowadays grown in significance due to increasing health and environmental concerns. For example, many developed cities worldwide are pushing towards better pollution monitoring infrastructure while biogas producers, such as landfill sites, are adopting stricter methane emission surveillance systems. The former is derived from the palpable deterioration of indoor and urban air quality as a result of the increasing number of combustion-based vehicles, and the fact that new medical studies continuously reveal the negative effects of such pollutants in human health [1]. The latter arises from the high global warming impact of $CH_{4}$ [2], and the considerable amounts of fugitive emissions that go undetected due to sparse measurement routines. This fact has triggered different governmental institutions, such as the Environmental Protection Agency of the United States of America (EPA), to search for and develop effective emission monitoring mechanisms [3].

In this regard, mobile robotics can indeed contribute with systems tailored for the automation of emission monitoring procedures [4]. Mobile robot olfaction (MRO), the branch of robotics that addresses the integration of gas and chemical sensors on-board mobile platforms [5], compromises systems that can acquire measurements at a high spatio-temporal resolution without exposing operators to dangerous environments, where, for example, high concentrations of airborne and chemical contaminants can be present. Moreover, the computational capabilities of MRO systems can be used, for example, to create maps of the distributions of gases [6,7,8,9,10], plan for full coverage surveillance tours [11], discriminate among different volatiles [12,13], or infer the location of gas leaks [14,15,16].

The development of MRO systems is not a trivial problem and, despite recent achievements, the potential of gas-sensitive mobile robots has yet to be fully realized. A key hurdle is the lack of proper datasets that can be used to conduct ground truth evaluations. This drawback originates from the great difficulty associated with performing experiments in real environments, and the current technological limitation to obtain ground truth information of the gases released and their distribution. Data collection is a complex and time-consuming process, even inside dedicated robot arenas as in [17]. The biggest challenge comes from the high complexity of the gas dispersion phenomenon, where many environmental and topological variables enter into play. Furthermore, this phenomenon is highly susceptible to small changes in these variables, often leading to substantial variations in the collected datasets. This makes repeatability between experiments hard to achieve.

It is thus of high importance to have proper evaluation tools to facilitate the development of new algorithms, sensors and platforms that comprise MRO systems. In this context, simulation tools with the capacity to properly handle the gas dispersion phenomenon can be used to perform extensive evaluations before moving to experimental trials in the real world. Different approaches have been developed and presented, ranging from simplistic models based on Gaussian-shaped plumes [18] to sophisticated fluid dynamics models that consider topological and environmental variables in the computation process [19]. However, most of these approaches do not consider sensing devices or mobile robots in the simulation, and while a few implementations of robotics-oriented gas dispersion simulation frameworks exist, they only consider simplified environments, are developed in outdated robotic software platforms or rely on expensive, external software packages.

In this work, we introduce GADEN, an open source gas dispersion simulation framework aimed at MRO related applications. GADEN not only simulates how gases disperse in any 3D environment (including obstacles), but also in mobile robotics platforms and sensing mechanisms. Concretely, the modularity of GADEN allows the user to implement or import different sensor models, integrate widely spread robotics algorithms for tasks such as localization, path planning or mapping, and to evaluate implemented MRO algorithms, all under a unified simulation framework. Specifically, the contributions that GADEN provides in the context of gas dispersion simulation for robotics related applications can be summarized as follows:

- Fully developed using the robot operating system (ROS) [20]. Arguably, ROS is the most widespread robotics OS used in academia and industry.
- Full support of 3D-CAD (computer-aided design models) to easily define complex environments (multiple rooms, obstacles, inlets/outlets, etc.).
- Relies on OpenFoam [21], an open source computational fluid dynamics toolbox, to obtain truthful wind fields within the 3D environment for a wide variety of wind conditions (Reynolds numbers, wind speeds, etc.).
- Implements the filament gas dispersion theory [22] over a time-evolving wind flow vector field to obtain accurate 3D gas distributions.
- Supports multiple gas-sources and different chemical substances simultaneously.
- Accounts for gravity and buoyancy forces by considering the molecular weight of the gases released.
- Simulates different gas sensing technologies, including metal oxide (MOX) and photo ionization (PID) gas detectors. Regarding wind flow measurements, a commonly used 2D ultrasonic anemometer is simulated. However, 3D airflow information is avaliable for the interested user.

GADEN is implemented as an ROS package, containing the different simulator components, manuals and test data. Both the code and the supplementary material (including a demonstration video) can be found at [23].

The different components of the simulator are described in detail in Section 4, after a thorough review of related works in Section 2, and the introduction of the framework architecture in Section 3. Then, in Section 5, we validate our proposal both quantitatively and qualitatively, by comparing our simulation results against a controlled real-world experiment (wind tunnel). Finally, we present some conclusions and future research lines in Section 6.

## 2. Related Work

Gas dispersion simulation (GDS for short) is a task that has been persistently addressed by different disciplines such as meteorology and computer science. Several taxonomies have been reported to classify the different approaches, for example, according to the spatial scale (i.e., macro and micro models) [24], or to the nature of the dispersion model [25].

According to the latter taxonomy, simplistic GDS models can be classified as box, Gaussian and analytical models. These models make strong assumptions regarding the underlying spatio-temporal properties of the gas distribution. For example, box models assume that gas dispersion can be discretized as a series of boxes of homogeneous gas concentration [26]. Gaussian models assume that gas dispersion follows a normal probability distribution [25]. Analytical approaches use empirical models regulated by a series of coefficients, which are set according to data from experimental campaigns [18,27]. Due to their simplicity, these models are computationally inexpensive, but not very precise in their estimations.

More sophisticated GDS approaches include the Lagrangian, Eulerian and Computational Fluid Dynamics (CFD)-based models. Lagrangian and Eulerian models simulate gas dispersion as the random walk of a large number of particles, affected by wind flow and turbulence fields. The most important difference between these two models is that Eulerian models employ a fixed three-dimensional Cartesian grid as a frame of reference rather than a moving frame, as in Lagrangian models. Their main disadvantage is the high computational cost when the number of particles increase [28,29]. CFD-based models, on the other hand, solve three dimensional equations for wind, temperature, humidity and gas concentrations [19]. These models are used for simulations that require a high spatio temporal granularity where physical obstacles, such as buildings and walls, are explicitly represented [30].

There exist several implementations of the above-mentioned GDS approaches in commercial and open source packages, for example, ANSYS [31] and OpenFoam (Section 1). While these software packages can produce sophisticated dispersion models, they do not allow for the possibility to simultaneously include robotic platforms and gas sensing mechanisms. In that sense, different frameworks have been proposed to combine mobile robotics simulators and GDS (see Table 1). Plumesim, introduced by Cabrita and co-authors [32], is an example of simulation framework oriented towards robotics applications. Plumesim is based on Player/Stage [33], and it allows the simulation of gas dispersal employing simplistic theoretical models such as Gaussian plumes [18], measurements acquired in real world experiments, or using external data generated with CFD tools. While Plumesim is a flexible simulation tool, it is limited by the very simplistic models used for gas dispersion, the lack of support for obstacles, and the fact that, when using CFD data, the dispersion of the chemical volatiles must also be computed by the external CFD tool, notably increasing the complexity of the simulation and requiring some CFD expertise.

More recently, Monroy et al. proposed a GDS framework for MRO related applications [34]. The authors developed their framework in the OpenMORA [35] robotics operating system. As input, the framework employs gas dispersion snapshots computed with an external tool, being able to simulate dynamic dispersion of multiple gas sources. The main drawbacks of this simulator are that OpenMORA lacks the support provided by more widespread frameworks like ROS, wind data is not supported, and the dispersion of gases must be computed by external CFD tools, something that hinders the flexibility of the simulator (e.g., changing the location of a gas source requires re-computing all the CFD data, which is computationally expensive).

Later, a 3D gas dispersion simulation framework was presented in [36]. The framework, developed in ROS, has a modular structure that enables the simulation of gas sensing mechanisms, physical obstacles, and different chemical species. Gas dispersion is carried out using a filament based model and wind flow patterns are externally generated using fluid dynamics tools such as OpenFoam. The main differences from the gas dispersal simulator presented in this work is that it only supports one emitting source, the definition of the environment topology is not straightforward (not supporting CAD models), and the performance of the simulation is considerably reduced as the simulation time passes.

It must be noticed that none of the papers discussed above, nor GADEN, consider the disruptions caused by the mobile robot when traversing the simulating environment. The straightforward solution, running the CFD simulation in real time, is limited by the available computational power, not being a feasible solution in most practical scenarios. Nonetheless, recent works have focused on providing a real-time approximation to the problem by modeling the airflow disruptions caused by, e.g., quadcopters [37,38], and then integrating these disruptions into the previously simulated gas dispersion data. Though this approach seems appealing, the integration of these disruption models into gas dispersion and robotics simulation engines is still an open research question.

## 3. Structure of the Simulator

**Figure 1:**
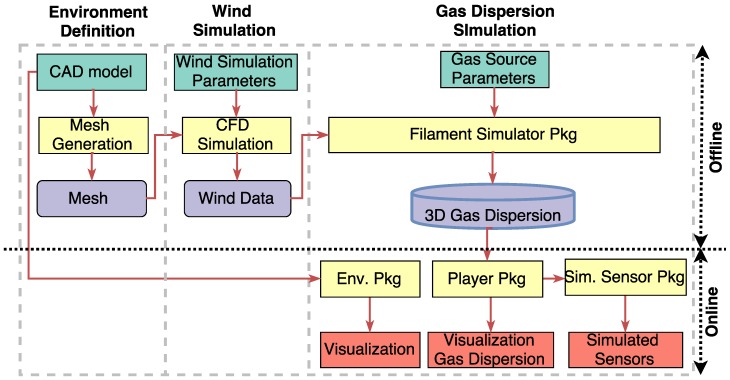
Block diagram of the presented gas dispersion simulator. Green, blue and red blocks represent input, intermediate and output data, respectively, while yellow blocks are processes.

The presented simulator follows a three-stages structure as depicted in Figure 1. In the first stage, the environment is defined by specifying the dimensions, obstacles and physical wind inlets/outlets among other parameters. Then, by means of CFD tools, the wind flow patterns within the environment are simulated, and finally, in the last stage, the estimation of the gas dispersion is computed by applying the filament dispersion model [22]. In the next sections, we describe all three stages to get an overview of the complete simulation process, and analyze the parameters and data types of each one.

### 3.1. Stage 1: Definition of the Environment

Along this work, the term “environment” refers to the set of walls, doors, windows, obstacles, and, in general, any item that may influence/disrupt the wind flows, and consequently, the gas dispersion. The accurate definition of the environment is one of the keys for a successful simulation of the wind flows by means of CFD tools (see Section 3.2). These numeric tools usually require the environment to be provided in a very specific and complex format that satisfies certain criteria in order to ensure a valid, and hence accurate, numeric solution. For example, OpenFOAM defines the environment as a “polyMesh”, a mesh of arbitrary polyhedra, bounded by arbitrary polygonal faces. This offers a big deal of freedom for the mesh generation and manipulation, something crucial when the geometry of the domain is complex or changes over time.

Previous approaches defined the environment manually (i.e., typing the polyMesh by hand, specifying the vertex and edges of the different elements of the mesh), and, consequently, the presented environments were very simplistic (usually a rectangle with a couple of obstacles in it). To overcome this limitation and enable the simulation of the gas dispersion within realistic environments, in this work, we rely on automated tools for the generation of the polyMesh from elaborated CAD.

**Figure 2:**
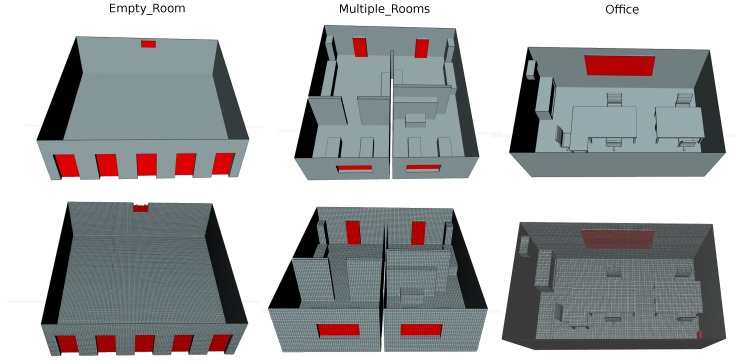
Three simulated environments with increasing complexity: An empty room, a multiple-room environment, and an office with tables and chairs. (**top row**) CAD models, (**bottom row**) meshes generated for their use in OpenFoam. Candidate inlets/outlets are marked in red.

As an illustration, Figure 2 shows three different environments designed with increasing complexity and their respective meshes ready to feed in the CFD wind simulation. The three scenarios represent an empty room with a large number of doors (inlets) and windows (outlets), a multiple room environment with basic furniture, and an office-like room with detailed objects such as tables and chairs.

### 3.2. Stage 2: CFD Wind Simulation

For the simulation of the wind flow, we rely on CFD tools. More concretely, we employ OpenFOAM, arguably one of the most widespread and active open-source CFD projects. OpenFoam takes as input the polyMesh of the environment, together with an extensive list of parameters that control the simulation process, such as boundary wind conditions, transient or steady simulation, turbulence level (i.e., Reynolds number), material properties, solver, etc. In general, non-experts in fluid dynamics may find this software too complex and with too many parameters, leading to unsuccessful simulations in many occasions. To relax this drawback, different graphical tools have appeared in the last years to control OpenFoam from a more user-friendly interface. This is the case, for example, of SimScale (used along this work for the generation of the CFD wind flow data), a web-based tool which allows importing the environment as a CAD model, generating the mesh required by OpenFOAM, and configuring the most important parameters of the CFD simulation through an easy and intuitive interface.

**Figure 3:**
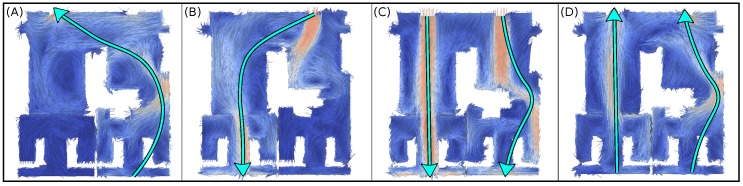
Four different wind simulations (**A**–**D**) for the multi-room environment as a result of different OpenFoam parameters. In all cases, a “slice” filter (at 0.5 m from the floor to appreciate the obstacles) has been applied to show only a 2D plane of the environment. Vectors represent the direction of the wind at each point, while the color represents the wind strength. Light-blue arrows depict the main wind flow direction within the environment. In all cases, wind strength at the inlets has been set to 1 m/s.

Figure 3 displays the results of three different CFD wind simulations for the multiple-rooms environment (in Figure 2), modifying the inlets/outlets, the wind characteristics or the turbulence model applied. As can be observed, the resulting wind vectors within the environment are highly dependent of the obstacles, presenting vortexes and complex wind structures which illustrate the intricacy of real wind-flows.

### 3.3. Stage 3: Gas Dispersion Simulator

Once the environment has been defined, and the simulation of the wind flow patterns has been generated, we can proceed to the 3D gas dispersion simulation. This stage, which represents the core contribution of this work, is done by a C++ implementation of the filament-based atmospheric dispersion model [22]. The main reasons behind the election of this specific dispersion model (see Section 2 for an overview of different models) are that it represents a good compromise between complexity and computational power, it is designed to replicate both the short-term and long-term exposure statistics of a chemical evolving in a turbulent flow, and it does not rely on a mathematical plume model, but is based on modelling different physical phenomenons that occur during gas dispersion. All these characteristics make this dispersion model ideal for the complex environments usually found in robotics applications, enabling time-variant chemical dispersion and accounting for the presence of obstacles. However, it must be stated that it does not account for temperature driven gradients or gravity forces, among other advanced parameters that may influence the gas dispersion.

**Figure 4:**
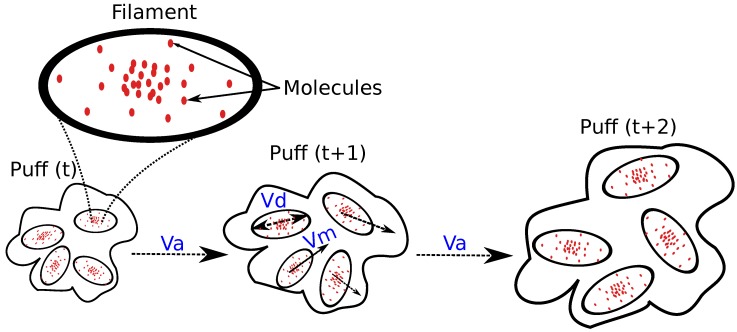
A gas release is modeled as a sequence of puffs, each one composed of multiple filaments (being a filament a 3D Normal distribution of gas molecules). Puffs are affected by advection and diffusion, altering the location of filaments by effect of the wind (v${}_{a}$) and random processes (v${}_{m}$), as well as their size (v${}_{d}$).

The filament dispersion model states that a chemical release can be represented as a sequence of puffs, each one composed of *n* filaments, and each filament being modeled as a 3D Normal distribution of molecules (see Figure 4). The puffs are manipulated by turbulent and molecular diffusion, while, at the same time, are being transported advectively by wind. Three dispersion phenomena are modeled according to the relative size of the puff with respect to the wind eddies as follows:

- **Eddies larger than the puff transport it as a whole**. This effect is modeled as an advective flow over the filaments, updating their location according to the local wind conditions (obtained from the CFD wind simulation).
- **Eddies on the order of the puff size cause significant growth/distortion of it**. This can be seen as a diffusive effect over the filaments of a puff, and is modeled as a random white noise affecting their location.
- **Eddies smaller than the puff mix the components of the puff, causing little puff motion or growth**. This represents the diffusion component of the molecules within a filament, being modeled as a continuous but slow growth of the filament size with time.

**Figure 5:**
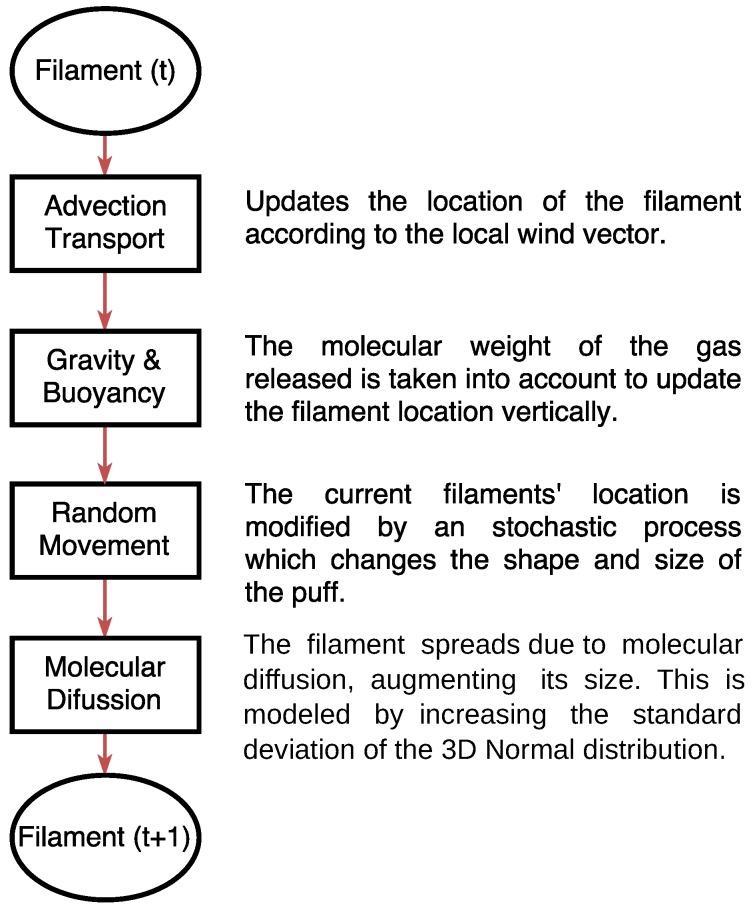
On each iteration, the location and shape of filaments are updated according to four dispersion phenomena: advection, gravity, randomness and diffusion.

On each iteration, the simulator updates the location of released filaments according to the above phenomena to estimate the new gas dispersion in the environment. Figure 5 illustrates the process, where it can be observed that a fourth dispersion effect is also considered. The latter accounts for gravity and buoyant forces, which, according to the chemical released, make the filaments decay or rise with respect the wind field. This is important since the filament model as described in [22] does not consider molecular weight, and, therefore, unrealistically, all gases behave in a similar way.

## 4. Implementation: The ROS-pkg

The presented gas dispersion simulator has been implemented as a ROS (Robotic Operating System) meta-package, and coded in C++. The reason behind implementing the software as an ROS package (ROS-pkg) instead of as an independent C++ software is to ease its integration with many other ROS-implemented modules (e.g., RVIZ [39] for visualizing the data, NDT-MCL [40] or RF2O [41] for robot localization, or artificial olfaction packages such as the KernelDM+V [42] or the GMRF [43] packages for gas distribution mapping). Concretely, the software has been organized in four packages that are described in detail below.

### 4.1. Filament Simulator Package

This ROS-pkg is the core of the gas dispersion simulator. It handles the movement of the puffs and filaments at each time-step, as well as estimates the gas concentration according to the location and shape of active filaments. Among its principal tasks, we highlight:

- Creates new filaments based on the 3D location of the gas source, the gas type and release rate.
- Updates the location of filaments according to the local wind conditions, the nature of the chemical and the structure of the environment (i.e., boundaries and obstacles).
- Removes filaments that reach the outlets defined in the simulated environment, avoiding the undesired accumulation of the gas concentration at those locations, and consequently, providing a more realistic simulation. It must be stressed that this feature is not considered by any of the previous GDS frameworks.
- Estimates the gas concentration at every point in the environment (3D grid-map) from the filaments present and their current shape and size.

The execution of these tasks can be computationally expensive; therefore, this package is designed to run offline, recording at each time-step the current 3D map of gas concentrations to file.

### 4.2. Environment Pkg

The objective of this package is to visualize the environment. That is, in order to plot the estimated gas concentration in the appropriate context, this package loads the CAD model of the environment and translates it to the RVIZ tool for visualization purposes.

### 4.3. Player Pkg

This package is in charge of loading the 3D gas concentration gridmaps produced by the “filament simulator pkg” and making such data available to the ROS architecture at every time-step. Hence, it is responsible for the visualization of the gas concentration as a point cloud, and, moreover, it implements two independent services to provide the gas concentration or the wind vector at a given 3D location.

**Figure 6:**
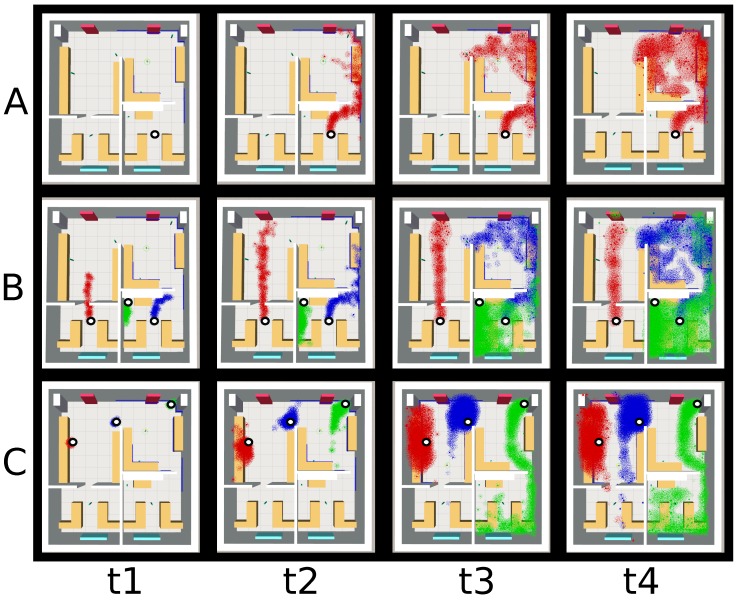
Snapshots of the gas dispersion at four time-steps (**t1**–**t4**) for three different scenarios (**A**–**C**) varying the wind flow, number and location of the gas sources. (**A**) when only one gas source is present and the wind flows from bottom to top (see Figure 3D); (**B**) introducing a total of three gas sources under similar wind flow conditions; and (**C**) changing the main wind flow from top to bottom (see Figure 3C) and setting three different gas sources. To allow an easy differentiation of the multiple gases, each one has been colored differently.

Since this package has basically no computational burden, it can run online, being only limited by the size of the environment and the desired refresh rate. Moreover, it facilitates loading multiple gas concentration gridmaps simultaneously, enabling the consideration of multiple gas sources and different chemical species. Figure 6 shows different gas dispersion simulations for the multi-room environment, varying the wind conditions, the gas source location, or the number and type of chemicals released.

### 4.4. Simulated Sensor Pkg

Finally, to exploit the gas dispersion simulator, we provide different sensor emulators that request the “Player pkg”, the value of the gas concentration or the wind vector, and simulate the output that such a sensor will provide. Currently, three sensor technologies have been implemented:

- **MOX**—Metal oxide gas sensors are one of the most common (and widely spread) technologies for robotic olfaction. These sensors are very sensitive, but lack selectivity, so the simulated behaviour should consider the presence of multiple gases. Concretely, we model these sensors following the manufacturer data-sheet to estimate the resistance ratio RS/R0 from the ground truth concentration provided by the simulator. Then, we apply a low pass filter to simulate the rise and decay response times, and finally we estimate the sensor resistance RS at time *t* given the reference resistance R0.
- **PID** Photo ionization detectors are another type of gas sensor which are very reliable, provide gas concentration in ppm units, and show a nearly immediate response. However, this type of detector responds to all gases with an ionization potential below the ionization energy of its lamp. That is, they cannot be used to classify or recognize the gas being detected, but only to estimate the concentration, given that the gas type is known. However, the concentration measurement delivered by these devices is a value proportional to the real gas concentration by a factor that depends on the gas type. Concretely, we have modelled the response of an 11.7 eV PID detector (RAE Systems Inc, San Jose, CA, United States), providing as output the weighted sum of gas concentrations from all gas sources present in the environment. The weight factor applied to each gas has been extracted from the RAE TN-106 technical specification [44].
- **Anemometer** GADEN also includes an ultrasonic anemometer emulator to analyze the direction and strength of the wind at any given location. Concretely, it models a 2D anemometer, which outputs the wind velocity and upwind direction. In our current implementation, measurement noise or confidence intervals of the anemometer are not modeled.

## 5. Validation

To provide qualitative and quantitative values of the accuracy of the proposed simulator, we present in this section a comparison between real olfaction data and that provided by GADEN. Concretely, we employ the dataset presented in [45], an extensive set of olfaction experiments with a chemical detection platform consisting of 72 MOX gas sensors in a wind tunnel test-bed facility. This dataset is excellent for our purposes because it not only provides an extensive set of data (18,000 time-series), but also parameters such as the wind speed, the environment dimensions and the gas source emission rate, parameters that are mandatory to obtain a realistic simulation that can be used for comparison.

**Figure 7:**
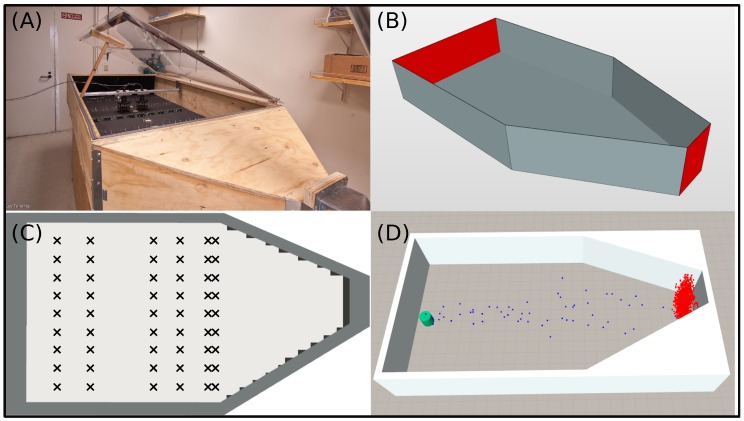
The wind tunnel test-bed facility: (**A**) picture of the physical wind tunnel used to collect the dataset (Image courtesy of Jordi Fonollosa), (**B**) illustration of the CAD model used during simulation (red faces corresponds to the wind inlet/outlet), (**C**) schema of the 54 e-nose locations used along the dataset, and (**D**) snapshot of a simulated gas plume (blue dots represents active filaments, while red dots are filaments that already passed through the outlet).

The dataset presents time-series for a wide variety of parameter configurations (wind speed, gas type, temperature modulation, etc.). From it, we selected a subset of data to compare with, corresponding to a methane gas source emitting at a concentration of 1000 pp and considering three different airflow speeds namely 0.1 m/s, 0.21 m/s and 0.34 m/s. Furthermore, we restrict the data to only one gas sensor (TGS-2620), but include all 54 locations of the e-nose within the wind tunnel. The simulation parameters are thus selected in order to follow the actual experimental setup as close as possible. For example, the concentration level at the gas source location is used to determine the number of filaments in the simulation. Figure 7 shows a picture of the wind tunnel, the respective CAD model used during simulation, a detailed view of the e-nose locations within the environment, and a snapshot of a simulated gas plume.

To compare the simulator results and measurements acquired in the wind tunnel, we compute probability density functions (PDF) at each of the 54 locations where gas measurements are recorded. It must be stressed that, for the real experiments, data was collected with different e-noses. Although each nose comprises the same number of sensors and the same sensor models, variations between sensors of the same model are expected due to e.g., sensor age and intrinsic differences. Moreover, the experiments in the wind tunnel were conducted during different periods of time, and, therefore, drift due to environmental conditions can also be expected. In order to minimize the drift effects on the sensor responses, we performed differential baseline correction over normalized measurements (between 0 and 1) acquired in the wind tunnel. Differential baseline correction is conducted as follows:

(1) $$x_{i}^{d}=x_{i}^{raw}-x^{0}.$$

In the above equation, $x_{i}^{raw}$ is the instantaneous measurement acquired at time *i*, $x^{0}$ is the baseline sensor response and $x_{i}^{d}$ is the baseline corrected response. To estimate the baseline response for each sensor, we averaged the measurements collected during 10 s, when the sensor is not exposed to any gas concentration.

**Figure 8:**
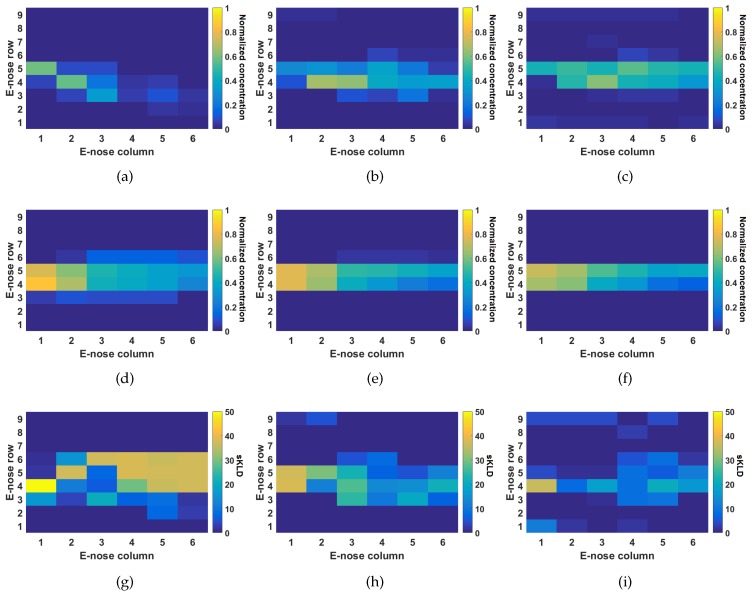
Comparison results between actual data collected in a wind tunnel and simulations. The experiments and simulations were conducted under different airflow profiles. (**a**,**d**,**g**) correspond to 0.1 m/s. (**b**,**e**,**h**) to 0.21 m/s. (**c**,**f**,**i**) correspond to 0.34 m/s. (**a**–**c**) correspond to actual measurements. (**d**–**f**) to simulated concentrations. (**g**–**i**) correspond to the symmetric Kullback–Leibler Divergence (sKLD) as explained in the text.

The actual and simulated concentration levels registered by the e-noses are shown as checkerboard plots in Figure 8a–f. Each e-nose corresponds to a single cell in the checkerboard plot. The color of the cell is determined by the normalized gas concentration (between 0 and 1). Notice that, for wind speeds of 0.21 m/s and 0.34 m/s, the simulator correctly predicts a laminar wind flow at the center of the wind tunnel, with concentrations that fairly match the measurements reported by the sensors in the e-noses. In the case of the simulations and the experiments conducted with a weak airflow (0.1 m/s), notice that the data from the e-nose sensors show a plume that menders towards the lower wall of the wind tunnel. This is likely to be the product of turbulence caused by thermal gradients, or by small imperfections in the tunnel construction, for example. Such complex turbulent behaviour was not simulated with the fluid dynamics tool (i.e., OpenFoam) and thus the simulation of 0.1 m/s shows a stable laminar plume as shown in Figure 8d.

Considering the above scenarios, we conducted a numerical comparison between the simulated plumes and the actual measurements from the e-noses. In order to do so, we computed the symmetric Kullback–Leibler divergence (sKLD), which is a widely used measure for the distance between probability density functions and has a low sensitivity to outliers. The sKLD is computed at each e-nose position as follows:

$$\begin{array}{ccc} KLD(M^{1}||M^{2}) & = & \sum_{i\in M}M_{i}^{1}\log\frac{M_{i}^{1}}{M_{i}^{2}},\\ sKLD(M^{g}||M^{w}) & = & KLD(M^{g}||M^{w})\\ & + & KLD(M^{w}||M^{g}), \end{array}$$

where $M^{w}$ is the PDF of the collected data at a given position of the e-nose, $M^{g}$ is the PDF computed from simulated data at the same position. Each PDF is computed using a histogram of 10 bins. Checkerboard plots that show the distribution of the sKLD for each e-nose position are shown in Figure 8g–i. Notice that, in the case of strong airflow (0.34 m/s), a good match between simulated data and real world data is observed. Similarly, simulation and data collected under an airflow of 0.21 m/s have a fair degree of similarity, denoted by cells colored with blue/light green shades. A few cells with High sKLD distances are observed on those e-noses that did not show any response to the gas concentration (bright shades of yellow). In the absence of a dominant airflow (0.1 m/s conditions), considerable differences between simulations and real-world data are observed (cells colored in yellow shades). This, as previously explained, is likely due to the fact that, in the real-world experiments, the plume meanders towards the lower wall of the wind tunnel, causing, in this way, low to zero exposure on those e-noses in the center right location of the wind tunnel.

Finally, to qualitatively evaluate GADEN and visualize the potential of the presented framework, we encourage the reader to watch the demonstration video where different environments and dispersion scenarios are shown. Both the video and the code, which is available as a ROS package, can be found at [23].

## 6. Conclusions

In this work, we present GADEN, a gas dispersion simulator that is aimed at robotics applications. GADEN is a modular tool developed in ROS that allows for creating sophisticated simulations that can be used used to validate, for example, Mobile Robotics Olfaction (MRO) algorithms. While several gas dispersion simulation engines exist, GADEN provides several improvements over the state-of-the-art. First, GADEN is fully developed in ROS, which allows for integrating robotics algorithms into the simulation engine. In this way, gas sensing and robotics algorithms, such as navigation and 3D perception, can be integrated and validated concurrently.

Second, GADEN provides an integrated solution to import sophisticated 3D-CAD models of the environment. In comparison, existing simulation tools allow for defining geometrical models from the environment, but only as simplistic models, where only a few cube-like obstacles might be present.

Third, GADEN allows for simulating environments where multiple gas sources of different analytes can be present simultaneously. This is particularly useful to validate algorithms for gas discrimination and the mapping of different chemical compounds.

Fourth, GADEN’s dual architecture, namely, the online and offline components, allow for easing off the computational demands of gas dispersion simulation. Existing simulation engines, on the other hand, compute gas dispersion as the simulator is being executed. The drawback of this approach is that, as the simulation time progresses, the computational demands increase substantially, limiting, in this way, the usability of the simulator.

In order to perform a quantitative evaluation of GADEN, we used real-world data acquired inside a wind tunnel, where a single emitting ethanol gas source was placed and an array of MOX sensors collected measurements. We thus created a CAD model of the wind tunnel and performed simulations under different airflow conditions. We compared the real-world measurements against simulated measurements at the sensor’s positions using the symmetric Kullback–Leibler divergence (sKLD). Our comparison shows a high degree of similarity between simulated and actual measurements when an advective airflow is present. When gas dispersion is dominated by turbulence, simulated and real-world measurements show higher divergence. This result is expected due to the the stochastic nature of turbulence, which is not considered in the gas dispersion simulation approach presented in this work.

Future research includes modeling the disruption caused by the mobile robot in the gas dispersion, as well as the automatic generation of the CAD models defining the simulation environment by integrating/developing tools that could obtain such models from dense point clouds, e.g.,employing RGB-D cameras or 3D lidars. These features would reduce the setup time and improve accuracy, providing a more realistic simulation environment. Furthermore, given the open source nature of this project, we encourage the community to use and contribute with new sensor models and further improvements. The code, user guides (from CAD support to ROS package configuration), as well as different datasets of simulated data are available at [23].

## Figures and Tables

**Table 1:** Comparison of Gas Dispersion Simulators with ROS support. In the table, MOX stands for Metal Oxide Sensors, PID stands for Photo Ionization Detectors, CAD stands for Computer Aided Design while CFD stands for Computational Fluid Dynamics.

		PlumeSim	Orebro	GADEN
**Environment**	Dimensionality	2D	3D	3D
	Obstacles	no	yes	yes
	Input format	Manual	Occupancy Files	CAD
**Wind Simulation**	CFD support	yes	yes	yes
**Gas Dispersion**	Model	CFD	Filament Model	Filament Model
	Gas sources	Only one	Only one	Multiple
	Chemical type	Independent	Gravity & Buoyancy	Gravity & Buoyancy
**Sensor Emulators**	Chemical	MOX	MOX	MOX, PID
	Anemometer	-	-	Ultrasonic
